# SADS-CoV NS3 induces apoptosis by blocking the formation of Bcl-xL-BAK complex

**DOI:** 10.1128/jvi.00216-26

**Published:** 2026-03-31

**Authors:** Chunxiao Mou, Meiqi Liu, Yingjie Xiang, Changhua Lin, Shengbin Zhang, Kaichuang Shi, Shuping Feng, Jingyun Ma, Zhenhai Chen

**Affiliations:** 1 College of Veterinary Medicine, Yangzhou University 38043, Yangzhou, Jiangsu Province, China; 2 Jiangsu Co-Innovation Center for Prevention and Control of Important Animal Infectious Diseases and Zoonoses, Yangzhou University 38043, Yangzhou, Jiangsu Province, China; 3 Guangxi State Farm Yongxin Husbandry Group of Xijiang Co., Ltd, Guigang, Guangxi Province, China; 4 Guangxi Center for Animal Disease Control and Prevention 117867 , Nanning, Guangxi Province, China; 5 College of Animal Science, South China Agricultural University 12526, Guangzhou, Guangdong Province, China; University of Kentucky College of Medicine, Lexington, Kentucky, USA

**Keywords:** SADS-CoV NS3, mitochondrial apoptosis, Bcl-xL, viral replication, viral pathogenicity

## Abstract

Swine acute diarrhea syndrome coronavirus (SADS-CoV) is an emerging porcine coronavirus with characteristics of bat origin, posing a potential threat to animal welfare and public health. Recent studies have shown that apoptosis induced by SADS-CoV contributes to virus replication. However, the virus proteins involved in SADS-CoV-induced apoptosis and their potential molecular mechanisms remain largely unknown. This study found that the SADS-CoV accessory protein NS3 interacted with the BH3 domain of Bcl-xL in the mitochondria. This interaction disrupted the Bcl-xL-BAK complex, leading to increased activation of BAK and subsequent induction of mitochondrion-mediated apoptosis. Furthermore, knockout of Bcl-xL reduced the NS3-induced apoptosis and increased the replication of SADS-CoV. Additionally, deletion of the NS3 gene decreased SADS-CoV-induced apoptosis and diminished the viral pathogenicity in murine models, indicating that NS3 is a critical determinant of SADS-CoV pathogenicity. In conclusion, our findings firstly reveal the mechanism by which SADS-CoV NS3 regulates apoptosis to enhance the viral replication and pathogenicity.

**IMPORTANCE:**

Swine acute diarrhea syndrome coronavirus (SADS-CoV) has caused significant disruptions in porcine breeding and raised concerns regarding potential human infection. Thus far, the roles of virus proteins in virus replication and pathogenesis remain largely unknown. Here, we first investigated the functions of SADS-CoV NS3 in apoptosis and viral pathogenicity. Our findings indicated that NS3 blocked the combination of anti-apoptosis protein Bcl-xL with pro-apoptosis protein BAK, then promoting the BAK pore-forming and inducing mitochondrion-mediated apoptosis, thereby enhancing the pathogenicity of virus. Moreover, we also discovered for the first time that Bcl-xL could act as an important antiviral factor to inhibit SADS-CoV replication. These results contribute valuable insights into the novel roles of bat-borne coronavirus accessory proteins in viral replication and pathogenicity in potentially infected hosts.

## INTRODUCTION

Over the past two decades, coronaviruses have posed significant challenges to global public health because they frequently circulate among animal and human populations, leading to economic disruptions and catastrophic loss of life (1). The emergence of swine acute diarrhea syndrome coronavirus (SADS-CoV), a novel porcine coronavirus, was initially documented in southern China in February 2017 (2). Subsequent outbreaks of SADS-CoV occurred in 2018, 2019, and 2021, presenting an ongoing concern for livestock herds in regions such as Guangxi, Fujian, Henan, and Jiangxi (3). This trend suggests the potential for SADS-CoV to cause broader epidemics in the future. SADS-CoV originated from a bat HKU2-related CoV before it spilled into swine populations (4). The resulting SADS-CoV infection is characterized by symptoms such as acute diarrhea, vomiting, and elevated mortality rates in young piglets (4). This virus has a wide host range and exhibits robust replication in mammalian cells, particularly in primary human lung and intestinal cells, underscoring the potential for zoonotic transmission (5).

SADS-CoV is classified within the family *Coronaviridae* and the genus *Alphacoronavirus*. It is characterized as a positive-sense, single-stranded, enveloped RNA virus, possessing a genome approximately 27 kb in length. This genome encodes 16 nonstructural proteins, 4 structural proteins, and 3 accessory proteins (NS3, NS7a, and NS7b) (6). All coronaviruses possess accessory proteins, and the number of accessory proteins and their functions varies among the members of the coronavirus family (7). While accessory proteins are not essential for the replication of coronaviruses *in vitro*, they are crucial in modulating innate immune responses, viral proliferation, and pathogenicity.

Apoptosis is initiated through two distinct signaling pathways: the extrinsic and intrinsic pathways (8). The intrinsic apoptotic pathway, which is mediated by mitochondria, is activated by intracellular stressors, leading to mitochondrial outer membrane permeabilization (MOMP) (8). MOMP is primarily regulated by the B-cell lymphoma 2 (Bcl-2) family of proteins, which are classified into three categories: anti-apoptotic proteins, pro-apoptotic proteins, and BH3-only proteins (5). Intracellular stress signals induce conformational changes in the pro-apoptotic effectors BAX and BAK. Subsequently, BAX and BAK translocate, oligomerize, and form large pores in the mitochondrial membrane, thereby altering MOMP and facilitating the release of cytochrome C (Cyto C). In the cytosol, the apoptosome is assembled, comprising Cyto C, apoptotic protease activating factor-1, and pro-caspase-9, while the released apoptosis-inducing factor (AIF) translocates to the nucleus to promote DNA fragmentation. Anti-apoptotic proteins, such as Bcl-2, MCL-1, and Bcl-xL, function by binding and sequestering the pro-apoptotic pore-forming proteins BAX and BAK. This process prevents the activation of caspase cascades that culminate in apoptosis (9).

Coronaviruses, encompassing severe acute respiratory syndrome coronavirus (SARS-CoV), SARS-CoV-2, porcine transmissible gastroenteritis virus (TGEV), and porcine epidemic diarrhea virus (PEDV), have developed a variety of mechanisms to manipulate apoptotic pathways. For example, TGEV infection downregulates Bcl-2 expression and upregulates BAX expression, thereby promoting BAX translocation from cytoplasm to mitochondria and activating the mitochondrial-mediated apoptosis pathway (10). PEDV infection induces the translocation of apoptosis-inducing factor (AIF) from mitochondria to the nucleus, without triggering the release of mitochondrial Cyto C, thereby initiating the process of apoptosis (11). SADS-CoV is also reported to induce caspase-dependent extrinsic and intrinsic apoptosis (12, 13). The replication of SADS-CoV could be affected by the inhibitors of apoptosis, indicating that SADS-CoV-induced apoptosis contributes to viral replication (12, 13). However, the molecular mechanisms by which SADS-CoV induces apoptosis are still largely unknown.

In this study, we found that NS3 significantly induced apoptosis. The specific mechanisms by which NS3 induces apoptosis remain to be identified. Therefore, we investigated the pathways by which NS3 induced apoptosis and identified the apoptotic proteins that interacted with NS3. Furthermore, we examined the underlying mechanisms through which NS3 induced apoptosis via these apoptotic proteins. Additionally, we assessed whether NS3 affected SADS-CoV-induced apoptosis and viral pathogenicity. The findings of this research offer novel insights into the functions of the SADS-CoV accessory protein and its role in viral pathogenesis.

## MATERIALS AND METHODS

### Cells and viruses

ST, Vero, and HEK293A cell lines were obtained from the China Institute for Veterinary Medicine and cultured in DMEM with 10% FBS (Sigma). All cell lines were maintained in a CO_2_ incubator at 37°C. The SADS-CoV strain (MK994934.1) was kept at our laboratory.

### Reagents and antibodies

The Z-VAD-FMK (C1202), Z-IEHD-FMK (Y062547), Z-LEHD-FMK (Y062548), TUNEL Detection Kit (C1086), MitoProbe Assay Kit (C2005), Annexin V Apoptosis Detection Kit (C1062S and C1065S), and Mitochondria/Cytosol Fractionation Kit (C3601) were obtained from Beyotime. The Lipofectamine 3000 reagent was purchased from Invitrogen. TRIzol reagent (R401-01), Virus RNA Extraction Kit (RM401), HiScript III RT SuperMix (R323-01), and Taq Pro Universal SYBR qPCR Master Mix (Q712-02) were purchased from Vazyme. Trypsin was obtained from Gibco. Trypticase phosphate broth (TPB) was obtained from Sigma. The SABC-POD kit and the peroxidase substrate kit were purchased from BOSTER. CytoTox 96 kit and Cell Titer-Glo Luminescent Assay were purchased from Promega.

The SADS-CoV N protein antibody was prepared by our laboratory. The GFP (AE078), HA (AE008), BAX (A12009), Bcl-2 (A19693), Cyto C (A4912), HRP-conjugated goat anti-rabbit IgG (AS039), Alexa Fluor 549-conjugated goat anti-mouse IgG (lAS054), and HRP-conjugated goat anti-mouse IgG (AS003) antibodies were purchased from ABclonal. Caspase 3 (D3R6Y) (14220), Caspase 8 (1C12) (9746), Caspase 9 (9502), and Cleaved-Caspase 3 (10661) antibodies were purchased from Cell Signaling Technology. The antibody against Tomm20 (ab186735) was obtained from Abcam. Antibodies against Flag (F1804) and Flag-beads (M8823) were purchased from Sigma. The Bcl-xL (10783-1-AP) and Actin (66009-1) antibodies were obtained from Proteintech. The Alexa Fluor 488-conjugated goat anti-mouse IgG (A23210) was purchased from Abbkine.

### Construction of plasmids

The NS3 gene was amplified from SADS-CoV cDNA and inserted into the pCAGGS-3Flag vector. The HA-tagged porcine Bcl-2, Bcl-xL, BAK, BAX, or MCL-1 expression plasmids were maintained in our laboratory. Truncated fragments of the Bcl-xL gene were inserted into the pCAGGS-HA plasmid. These recombinant plasmids were identified by DNA sequencing. The primers used are listed in Table 1.

**TABLE 1:** Sequences of the primers used for PCR

Primer	Sequence
SADS-CoV-NS3 F	CGGGGTACCATGTTTGGTGGACTTTTC
SADS-CoV-NS3 R	CCGCTCGAGTCAACAATCTTCAGAAAC
SADS-CoV F1	CTGGTAGGCGGCCGCCTCATTCAAGCTTGTGGACTTTAATTAAGACAGATCCTAGGAGAGTAAGCTAGCGTGTCTAGACGTCG
SADS-CoV R4619	ATCTGTCTTAATTAAATAATGACCACAATCATCACC
SADS-CoV F4619	GGTCATTATTTAATTAATGACTACGACAAGAG
SADS-CoV R9916	CCAAAACATGCCTAGGGCAATAAACC
SADS-CoV F9916	GACAGATCCTAGGCATGTTTTGGCTAGTGACACTAC
SADS-CoV R18105	GTGTCATGAGCTCTAGCTTTAACTGGCTTGACAATG
SADS-CoV F18105	TAAAGCTAGAGCTCCACCGGGTGAAC
SADS-CoV R21496	ATTTACGGTGGATCCATAGCTCC
SADS-CoV F21496	TGACACGGATCCACCGTAAATGGGGTGTTAAC
SADS-CoV R27845	ACTCAGGTCGGACCGCGAGGAGGTG
pBAC-SADS-NS3 F	ACTTTTCCCATTGAGGAGGAGTT
pBAC-SADS-NS3 R	GTTGAGTTTCTTGCAGCTGAAAATTAG
S-GFP F	TTATGTCAAATGGGCTTGGTGGCA
S-GFP R	CCCTTGCTCACCATTATTGGACGTGGACCTTTTCAATCTC
GFP-NS3 F	TCCACGTCCAATAATGGTGAGCAAGGGCGAGG
GFP-NS3 R	TGTGTTAGTCACTTGTACAGCTCGTCC
GFP-E F	GGACGAGCTGTACAAGTGACTAACACA
GFP-E R	AGAGAACAAGTATCAACACTAAAAGCC

### Cell viability assay

The activities of lactate dehydrogenase (LDH) and adenosine triphosphatase (ATPase) were evaluated to determine cell viability. HEK293A cells were transfected with pCAGGS-3Flag-NS3. The cell supernatants were centrifuged for the LDH assay with the CytoTox 96 kit. Cell lysates were analyzed for ATPase activity using the Cell Titer-Glo Luminescent Assay, per the manufacturer’s instructions. The cell viability of ST-ΔBcl-xL or ST cells was similarly assessed using these methodologies.

### TUNEL assay

TUNEL assay kit was used to detect apoptosis in HEK293A cells transfected with pCAGGS-3Flag-NS3 or ST cells infected with SADS-CoV. The cells were fixed, permeabilized, and treated with the TUNEL reaction mixture. They were then stained with Flag-specific mAb or SADS-CoV N-specific mAb, Alexa Fluor 549-conjugated antibody, and DAPI, followed by confocal microscopy (Leica) analysis.

### Flow cytometric analysis

The apoptosis was examined by Annexin V Apoptosis Detection Kit. Cells were collected and washed by PBS, and then stained with Annexin V-FITC/propidium iodide (PI) or Annexin V-APC/PI. The percentage of apoptotic cells was detected via flow cytometry (BD FACSVerse). At least 10^5^ events were recorded for each data point. Data were analyzed using FlowJo V10 software.

### Western blotting analysis

Cells were lysed with buffer containing protease and phosphatase inhibitors. For protein resolution, samples were treated with 5× SDS loading buffer, boiled at 100°C for 10 min, and separated via SDS-PAGE. For non-denaturing PAGE, samples were mixed with 5× non-denaturing buffer and resolved in SDS-free gels. Proteins were transferred to PVDF membranes. Membranes were blocked by 5% skim milk in TBS-Tween for 2 h, incubated with primary antibodies overnight at 4°C, and treated with HRP-conjugated secondary antibodies. Finally, proteins were visualized using the ECL system (Tanon), and the ImageJ software was used to determine gray density.

### Cytosolic and mitochondrial fractionation

The cytosolic and mitochondrial fractions were separated via Mitochondria/Cytosol Fractionation Kit. HEK293A cells were transfected with pCAGGS-3Flag-NS3 for 24 h. The cells were collected, washed, and then resuspended in lysis buffer with protease inhibitors. Subsequently, the cells were homogenized with glass homogenizers, and the supernatants were centrifuged at 12,000 × *g* for 10 min at 4°C. The resulting supernatant was the cytosolic fraction, and the pellet was the mitochondrial fraction, obtained using mitochondrial lysis buffer.

### Analysis of the mitochondrial membrane potential

The mitochondrial membrane potential was detected using MitoProbe Assay Kit. Cells were incubated with 2 nM JC-1 dye and analyzed by flow cytometry at 529 nm (green) and 590 nm (red) emissions. A minimum of 10,000 events were recorded per data point and analyzed with FlowJo V10 software.

### Co-immunoprecipitation (Co-IP) assay

HEK293A cells transfected with the indicated plasmids were lysed with NP-40 lysis buffer. The supernatant was incubated with the indicated magnetic beads at 4°C for 8 h. After washes in NP-40 lysis buffer, the immune complexes were analyzed by immunoblotting.

### Immunofluorescence assay (IFA)

Cells were fixed in 4% paraformaldehyde, permeabilized with 1% Triton X-100, and blocked with 1% BSA. Subsequently, they were incubated with primary antibodies at 4°C overnight, and then incubated with fluorescence-conjugated secondary antibodies at 25°C. After the DAPI staining was performed, the fluorescence images were captured using a Leica microscope.

### Construction and analysis of ST-ΔBcl-xL cell line

The porcine Bcl-xL sgRNA was designed through the http://crispor.tefor.net/. The sequences of sgRNA targeting the first exon of Bcl-xL were F, 5′-CACCGTTTGAACTGAGGTACCGGA-3′, and R, 5′-AAACTCCGGTACCTCAGTTCAAAC-3′. To produce lentivirus for delivering sgRNA, HEK293A cells were co-transfected with LentiCRISPRV2-Bcl-xL-sgRNA, psPAX2, and pMD2.G. Lentivirus was collected and then used to infect ST cells to develop the ST-ΔBcl-xL stable cell line. The effect of Bcl-xL knockout was confirmed by Western blotting.

### Recovery of the NS3-deleted SADS-CoV

The SADS-CoV infectious clone plasmid (pBAC-SADS-CoV) has been successfully constructed at our laboratory. The plasmid included the CMV promoter at the 5′ end of the virus genome, while 35-residue poly(A) tail, HDV ribozyme sequence, and BGH termination signal were set at the 3′ end of the virus genome (Fig. 6A). To delete the NS3 gene in the virus genome, the pBAC-SADS-CoV-GFP/NS3 plasmid with the GFP gene replacing the NS3 gene was created through Red recombination in *E. coli* strain GS1783 (Fig. 6A), as previously described (14). Both the pBAC-SADS-CoV and pBAC-SADS-CoV-GFP/NS3 plasmids were confirmed by PCR and DNA sequencing.

Vero cells were transfected with pBAC-SADS-CoV or pBAC-SADS-CoV-GFP/NS3 and pCAGGS-SADS-CoV-N plasmids for 4–5 h. After washing the cells with PBS, they were cultured in DMEM maintenance medium containing 2–3 μg/mL trypsin and 10% trypticase phosphate broth (TPB). The cytopathic effect and green fluorescence were observed daily.

### Viral growth curves

Vero cells were infected with the SADS-CoV-wt, rSADS-CoV, or rSADS-CoV-GFP/NS3 at an MOI of 0.01. After a 2-h incubation, the cells were washed with PBS and cultured in the maintenance medium including 2–3 μg/mL trypsin and 10% TPB. Cell culture supernatants were collected at 12, 24, 36, 48, 60, and 72 h post-infection (hpi), and the TCID_50_/mL (Log10) was calculated using the Reed-Muench method.

### Mouse virus infection and pathogenesis study

To compare the virulence difference of SADS-CoV-wt and rSADS-CoV-GFP/NS3, SPF ICR mice aged 3 to 4 days were purchased from the Yangzhou University Comparative Medical Research Institute in Jiangsu, China. The mice were divided into three groups (*n* = 12) and infected with 2×10^4^ TCID_50_ of SADS-CoV-wt or rSADS-CoV-GFP/NS3 by oral (p.o.) or intraperitoneal (i.p.) injection. The control mice were treated with DMEM. The weights, mortality, and health conditions of all mice were observed daily for 21 d post-infection (dpi). At 14 dpi, three mice from each group were euthanized for viral titer measurement, histopathological examination, and cytokine analysis within the tissues.

### Histopathologic analysis

Initially, the tissue samples were fixed in a 4% paraformaldehyde, followed by dehydration, clearing with xylene, embedding in paraffin, and sectioning into slices of 4 μm thickness. Subsequently, the histological sections were dewaxed and rehydrated using a graded ethanol series. The sections were then subjected to hematoxylin staining for 5 min and differentiated with 1% hydrochloric acid alcohol solution. Thereafter, the sections were stained with eosin for 3 min, dehydrated using graded ethanol, treated with xylene, and finally sealed with neutral resin.

### Immunohistochemical staining

Paraffin sections were dewaxed, rehydrated, and underwent antigen retrieval in 10 mM citrate buffer at 95°C for 30 min. They were then blocked with 5% normal goat serum and incubated with Cleaved-Caspase 3 (1:100) overnight at 4°C. Negative controls used only PBST. Signal amplification and visualization were achieved using the SABC-POD and peroxidase substrate kits.

### RNA isolation and quantitative reverse transcription PCR

All collected mouse tissue samples were weighed, homogenized in PBS, and subjected to total RNA extraction, following the manufacturer’s guidelines. The reverse transcription PCR was performed using HiScript III RT SuperMix. The primers for quantitative fluorescence detection were designed on the basis of the conserved region of the SADS-CoV N gene. Fluorescence quantitative detection was performed via the Taq Pro Universal SYBR qPCR Master Mix on the CFX96 Real-Time PCR Detection System (Bio-Rad).

Total RNA from tissues was subjected to total RNA extraction, while viral RNA from SADS-CoV infected cells was extracted using a Virus RNA Extraction Kit. Subsequent reverse transcription PCR and fluorescence quantitative detection were conducted. The GAPDH gene was used as an internal reference. The mRNA levels of target genes were calculated using the 2^−△△Ct^ method. The primers used are listed in Table 2.

**TABLE 2:** Sequences of the primers used for qPCR

Primer	Sequence
SADS-CoV-N F	TTGTCACTCTTTGCGCCTTTG
SADS-CoV-N R	AGCATCTGCGTGAGGACCAG
Pig-GAPDH F	GTCGGAGTGAACGGATTTGGC
Pig-GAPDH R	CACCCCATTTGATGTTGGCG
Mouse-GAPDH	GAAATCGTGCGTGACATCAAAG
Mouse-GAPDH	TGTAGTTTCATGGATGCCACAG
Mouse-IL-6 F	TTCCATCCAGTTGCCTTCTTGG
Mouse-IL-6 R	TTCTCATTTCCACGATTTCCCAG
Mouse-IFN-γ F	ATGAACGCTACACACTGCATC
Mouse-IFN-γ R	CCATCCTTTTGCCAGTTCCTC
Mouse-IL-1β F	TGCCACCTTTTGACAGTGATG
Mouse-IL-1β R	AAGGTCCACGGGAAAGACAC

### Statistical analysis

Data are presented as mean ± SD and analyzed using one-way ANOVA with GraphPad Prism. Significance is marked by * for *P* < 0.05 and ** for *P* < 0.01; ns denotes not significant.

## RESULTS

### SADS-CoV NS3 facilitates mitochondrion-mediated apoptosis

**Fig 1:**
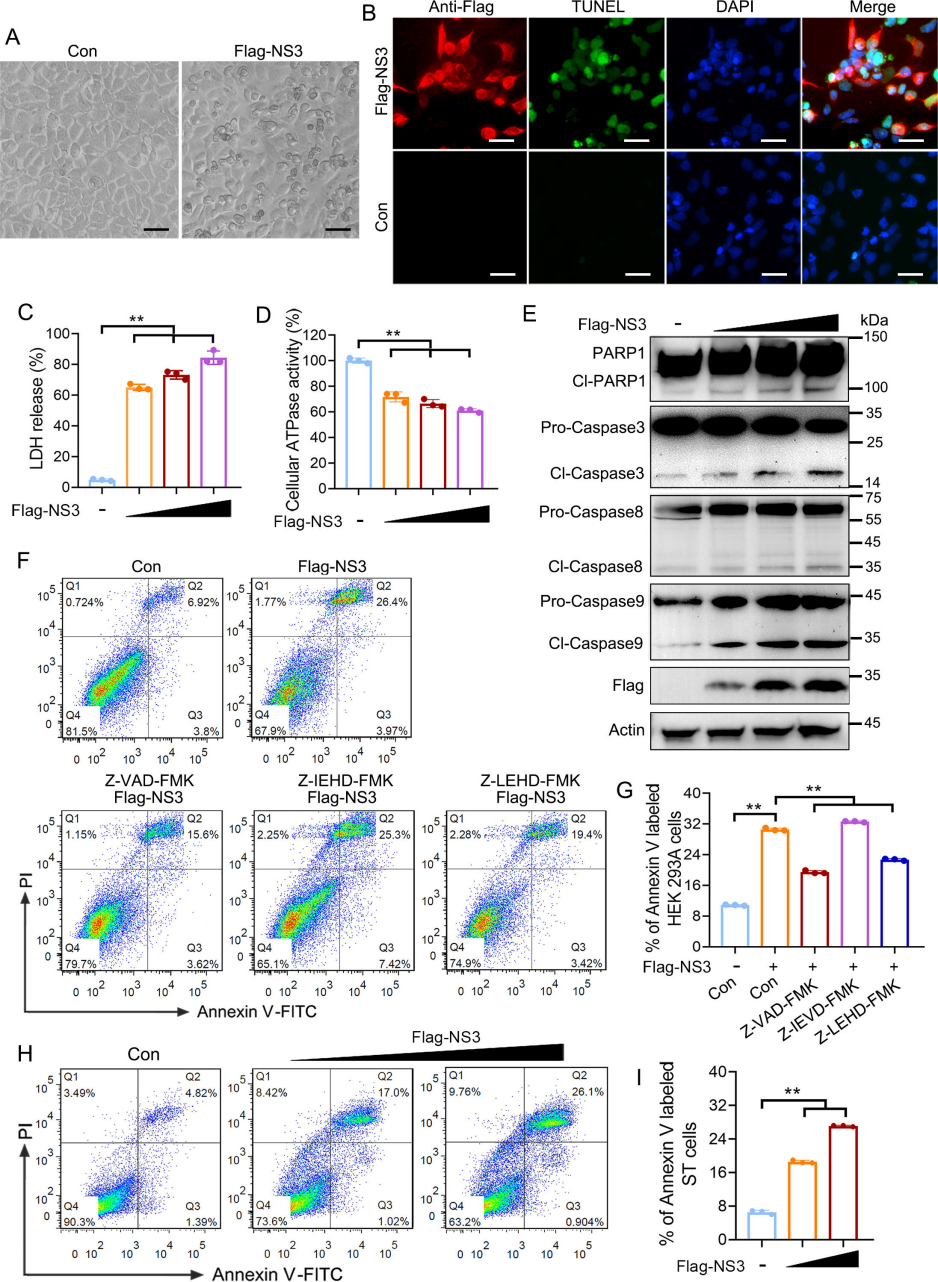
SADS-CoV NS3 induces mitochondrion-related apoptosis. (**A**) The plasmids expressing Flag-NS3 were transfected into HEK293A cells and the cytopathic effects were observed by microscope. (**B**) NS3 induced DNA damage in the HEK293A cells. Apoptotic cells were detected using TUNEL staining and analyzed using laser confocal microscopy. Scale bars, 100 µm. (**C and D**) SADS-CoV NS3 caused cell death in a dose-dependent manner. HEK293A cells transfected with plasmids expressing Flag-NS3 were analyzed for lactate dehydrogenase (LDH) levels in the supernatants (**C**) and cellular ATP enzymatic activity (**D**). (**E**) NS3 activated Caspase 3/9 to promote poly (ADP-ribose)-polymerase 1 (PARP1) cleavage. HEK293A cells were transfected with Flag-NS3, and then the activation of Caspase 3/8/9 and the cleavage of PARP1 in the cell lysates was determined using Western blotting. (**F and G**) The percentage of NS3-induced apoptosis in HEK293A cells. HEK293A cells pretreated with Z-VAD-FMK, Z-IEHD-FMK, or Z-LEHD-FMK were transfected with Flag-NS3. Cells stained with annexin V-FITC and propidium iodide (PI) were analyzed by flow cytometry, and the percentage of cells in Annexin V positive quadrant was quantified. (**H and I**) The percentage of NS3-induced apoptosis in ST cells. ST cells were transfected with Flag-NS3 in a dose-dependent manner. Cells stained with annexin V-FITC and propidium iodide (PI) were analyzed by flow cytometry, and the percentage of cells in Annexin V positive quadrant was quantified. The results shown are representative of three independent experiments (mean ± SD). **, *P* < 0.01.

Some coronavirus accessory proteins are involved in virus-induced apoptosis, affecting the pathogenicity of the virus. Under a microscopy, we observed that HEK293A cells overexpressing SADS-CoV NS3 exhibited apoptotic cell morphology (Fig. 1A). Furthermore, the TUNEL assay results showed that intracellular fragmentation of DNA was triggered in the NS3-overexpressing cells (Fig. 1B). Meanwhile, we observed that NS3 decreased the cell viability dose-dependently, as determined by quantifying lactate dehydrogenase (LDH) levels in the supernatants and cellular ATPase activity (Fig. 1C and D). These indicate that NS3 can induce apoptosis. Moreover, Western blotting showed that the expression of cleaved-Caspase 3/9 and DNA repair enzyme PARP1 robustly increased in the NS3-overexpressing cells (Fig. 1E). Flow cytometry analysis revealed that approximately 30% of the NS3-transfected cells underwent apoptosis, as indicated by Annexin V positivity (Fig. 1F and G). The broad-spectrum caspase inhibitors (Z-VAD-FMK), Caspase 8 inhibitors (Z-IETD-FMK), and Caspase 9 inhibitors (Z-LEHD-FMK) were used to further confirm the types of apoptosis induced by NS3. The results showed that in the Z-VAD-FMK and Z-LEHD-FMK pretreatment groups, the percentage of apoptotic cells induced by NS3 decreased by 11% and 7.5%, respectively, while the percentage of apoptotic cells in the Z-IETD-FMK pretreatment group did not change (Fig. 1F and G). In addition, NS3 was found to induce apoptosis in ST cells by flow cytometry analysis (Fig. 1H and I). These results indicate that NS3 can activate Caspase 3/9 to induce apoptosis.

**Fig 2:**
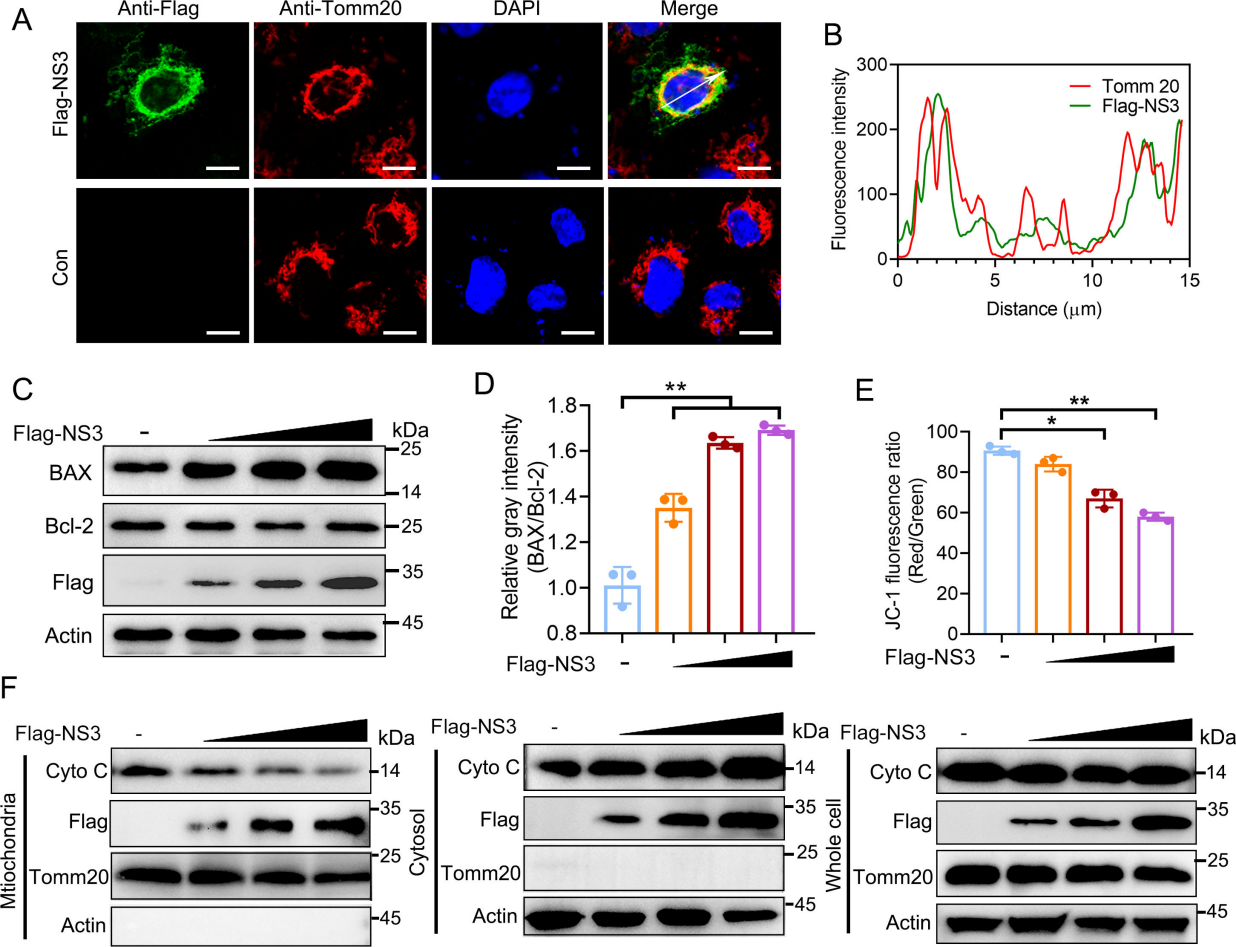
SADS-CoV NS3 localizes to mitochondria and induces Cyto C translocation. (**A and B**) SADS-CoV NS3 localizes to mitochondria. HEK293A cells were transfected with plasmids expressing Flag-NS3. The subcellular localization of Flag-NS3 (green) and Tomm20 (red) was observed using laser confocal microscopy. Scale bars, 50 µm. The fluorescence co-localization was analyzed by Image J. (**C and D**) NS3 increased the BAX/Bcl-2 ratio. The expression levels of BAX and Bcl-2 in HEK293A cells transfected with Flag-NS3 were determined using Western blotting, and the resulting BAX/Bcl-2 ratio is presented in the graph. (**E**) Detection of the mitochondrial membrane potential. HEK293A cells transfected with Flag-NS3 were harvested, stained with JC-1, and 10,000 cells were analyzed using flow cytometry. (**F**) NS3 induced Cyto C translocation. Cell lysates from HEK293A cells expressing Flag-NS3 were subjected to cytoplasmic and mitochondrial separations, followed by analysis of Cytochrome C translocation via Western blotting. Tomm20 and actin were used as internal controls for mitochondrial and cytosolic fractions, respectively. The results shown are representative of three independent experiments (mean ± SD). *, *P* < 0.05; **, *P* < 0.01.

Caspase 9 is recognized as a hallmark of intrinsic apoptotic pathways, which is also known as mitochondrial apoptotic pathway. Subsequently, we detected the subcellular localization of NS3 in HEK293A cells by confocal immunofluorescence assay. The NS3 was found to partially colocalize with the mitochondrial marker Tomm 20 (Fig. 2A and B), indicating that a part of NS3 localizes in mitochondria. Moreover, NS3 increased the BAX/Bcl-2 ratio (Fig. 2C and D), decreased the inner mitochondrial membrane potential (Fig. 2E), decreased the protein level of Cyto C in the mitochondrial fraction, and concomitantly increased the Cyto C in the cytosol (Fig. 2F). Taken together, the results show that SADS-CoV NS3 localizes to mitochondria and facilitates mitochondrion-mediated apoptosis.

### SADS-CoV NS3 interacts with Bcl-xL and disturbs BAK-Bcl-xL complex

**Fig 3:**
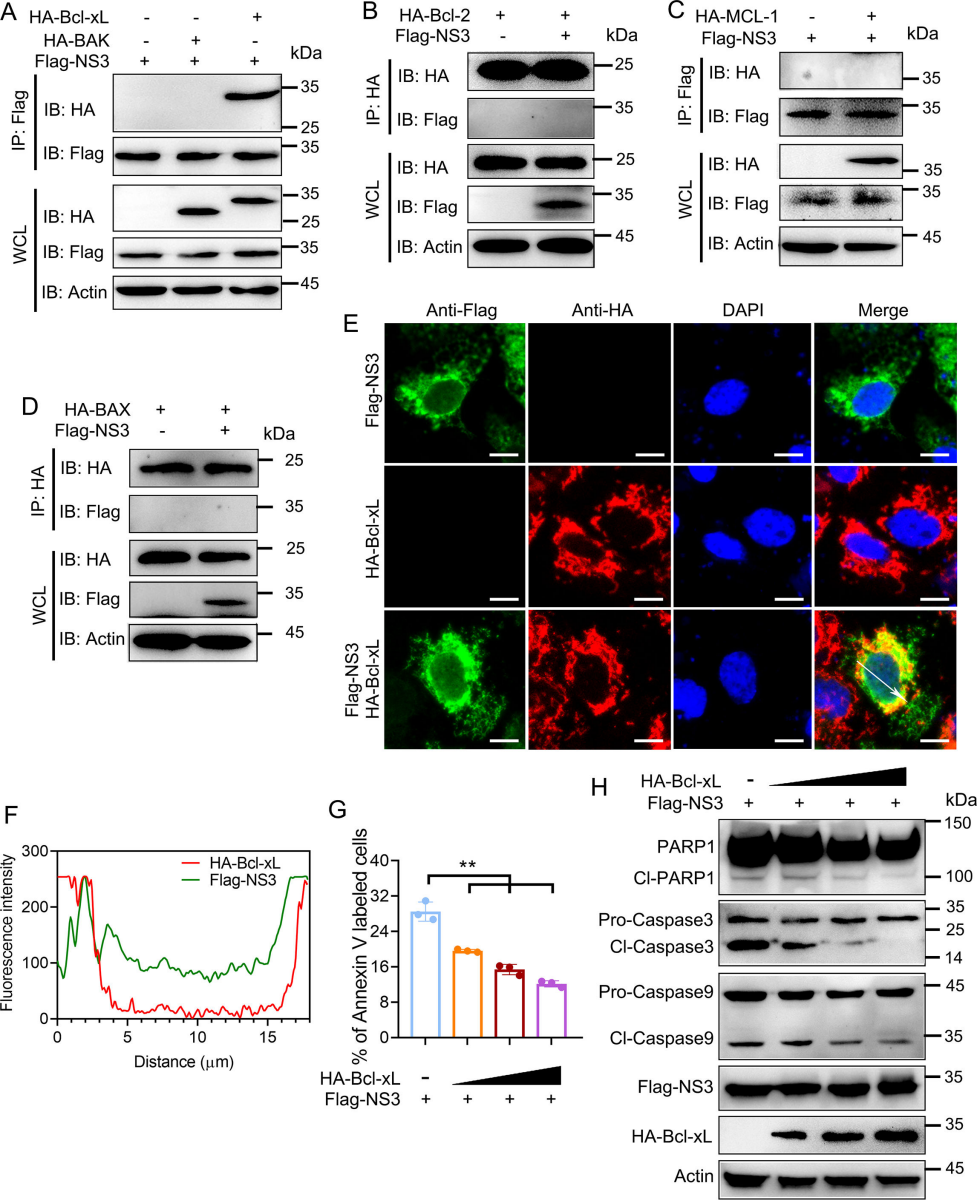
SADS-CoV NS3 interacts with Bcl-xL. (**A–D**) Interactions between proapoptotic or antiapoptotic proteins and NS3. The Flag-NS3 expression plasmid was co-transfected with HA-porcine Bcl-2, Bcl-xL, BAK, BAX, and MCL expression plasmids into HEK293A cells respectively, and the lysates were subjected to co-immunoprecipitation (Co-IP) analysis. (**E and F**) Co-localization of Flag-NS3 (green) and HA-Bcl-xL (red) was observed using laser confocal microscopy. Scale bars, 50 µm. The fluorescence co-localization was analyzed by Image J. (**G and H**) Bcl-xL overexpression decreased the NS3 induced apoptosis. The Bcl-xL expression plasmids were co-transfected with Flag-NS3 expression plasmid, and the apoptosis levels were assessed using flow cytometry (**G**). The cleavage of Caspase 3/9 and PARP1 was monitored via Western blotting (**H**). The results shown are representative of three independent experiments (mean ± SD). **, *P* < 0.01.

Mitochondrion-dependent apoptosis is regulated by members of the Bcl-2 family. Co-IP assays were performed to determine whether members of the Bcl-2 family are involved in NS3-induced apoptosis. After detecting the interaction between NS3 and porcine antiapoptotic proteins (Bcl-2, MCL-1, and Bcl-xL) and proapoptotic proteins (BAX and BAK), the results showed that NS3 did not interact with Bcl-2, MCL-1, BAX, or BAK, but was capable of interacting exclusively with Bcl-xL (Fig. 3A through D). Confocal immunofluorescence assay further showed that NS3 was highly co-localized with porcine Bcl-xL in HEK293A cells (Fig. 3E and F). In addition, while keeping the same NS3 protein input, the level of mitochondrial-dependent apoptosis in HEK293A gradually decreased with the increase in Bcl-xL protein input, as evidenced by a gradual decrease in the apoptosis level, Caspase 3/9 activations, and PARP1 hydrolysis (Fig. 3G and H). These indicate that Bcl-xL can inhibit NS3-mediated mitochondrial-dependent apoptosis.

**Fig 4:**
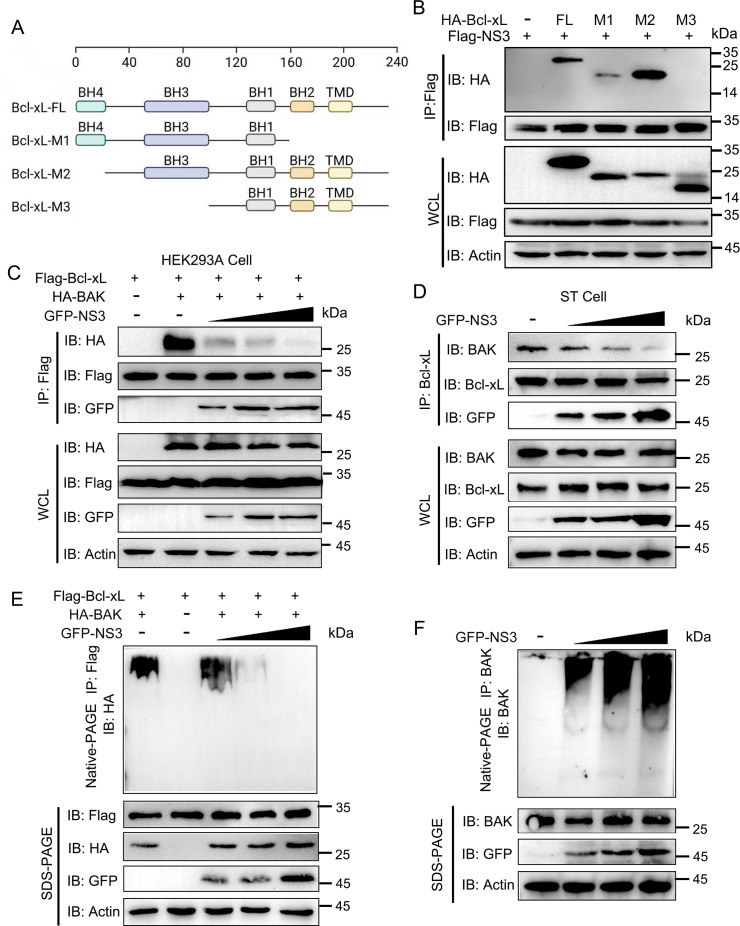
SADS-CoV NS3 disturbs the Bcl-xL-BAK complex. (**A**) Schematic domains of Bcl-xL and its truncation mutants. (**B**) The crucial region of Bcl-xL binding to NS3. Bcl-xL and its truncation mutant expression plasmids were co-transfected with the plasmid expressing Flag-NS3, and their interactions were detected by Co-IP analysis. (**C and D**) Competition of NS3 with BAK to bind to Bcl-xL. Plasmids expressing GFP-NS3 were co-transfected with Flag-Bcl-xL and HA-BAK expression plasmids in a dose-dependent manner in HEK293A cells. The cell lysates were subjected to Co-IP analysis using the anti-Flag beads (**C**). Similarly, plasmid expressing GFP-NS3 was transfected into ST cells for Co-IP analysis using the anti-Bcl-xL beads (**D**). (**E**) NS3 decreased the formation of Bcl-xL-BAK complex. The GFP-NS3 expression plasmids were co-transfected with Flag-Bcl-xL and HA-BAK expression plasmids into HEK293A cells in a dose-dependent manner, and cell lysates were subjected to detection via native-PAGE using the anti-Flag beads. (**F**) The formation of the BAK homo-oligomerization was detected by native-PAGE utilizing the anti-BAK beads.

Bcl-xL contains four conserved regions known as the Bcl-2 homology (BH) domains 1 to 4, as well as a hydrophobic C-terminal region, which is predicted to act as a transmembrane anchor. Hence, Bcl-xL was truncated to identify the crucial domain responsible for the interaction of Bcl-xL with NS3 (Fig. 4A). The results showed that NS3 specifically interacted with the BH3 region (56–100 aa) of Bcl-xL (Fig. 4B). Bcl-xL mainly combines with BAK, preventing BAK from accumulating at the mitochondrial membrane and subsequently leading to apoptosis. In this process, the BH3 region of Bcl-xL is important for the binding of Bcl-xL to BAK (15). Based on this, we speculated that NS3 may compete with BAK to bind Bcl-xL in the Bcl-xL-BAK complex, causing the release of BAK upon dissociation from the complex. To verify this, the HEK293A cells were transfected with Flag-Bcl-xL, HA-BAK, and GFP-NS3-expressing plasmids, and then Co-IP assays were performed to determine their interaction. The results revealed that NS3 blocked the interaction between Bcl-xL and BAK (Fig. 4C). Meanwhile, this inhibition was corroborated in ST cells through the detection of endogenous Bcl-xL and BAK (Fig. 4D). Furthermore, native-PAGE assays were used to examine the formation of BAK-Bcl-xL and BAK-BAK complexes in the HEK293A cells expressing GFP-NS3. The results indicated that NS3 led to a reduction in the formation of the BAK-Bcl-xL complex (Fig. 4E) while promoting the BAK homo-oligomerization (Fig. 4F). These findings suggest that NS3 competes with BAK for binding to Bcl-xL, causing the release of BAK from the Bcl-xL-BAK complex and inducing mitochondrial-dependent apoptosis.

### Role of Bcl-xL in SADS-CoV NS3-induced apoptosis

**Fig 5:**
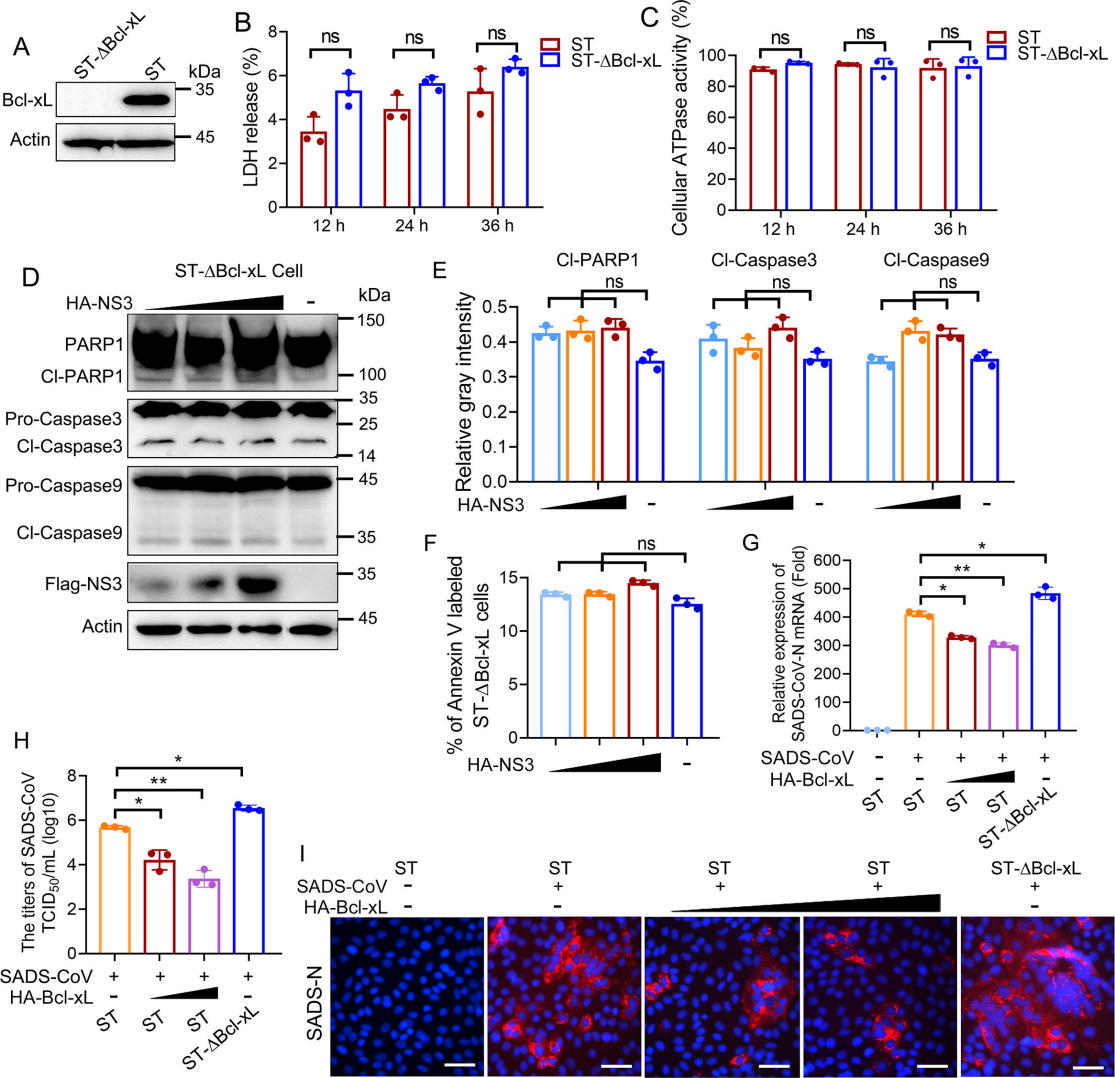
The role of Bcl-xL in NS3-induced apoptosis and the replication of SADS-CoV. (**A**) The knockout of Bcl-xL was identified by Western blotting analysis using an anti-Bcl-xL antibody. (**B and C**) The viability of the ST-∆Bcl-xL cells was assessed. The LDH levels in the supernatants and cellular ATP enzymatic activity in both ST cells and ST-∆Bcl-xL cells were analyzed. (**D–F**) Bcl-xL knockout disturbed the NS3-induced apoptosis. The NS3 expression plasmids were transfected into ST-∆Bcl-xL cells in a dose-dependent manner. The activation of Caspase 3/9 and the cleavage of PARP1 were assessed through Western blotting (**D**), with their relative gray intensities normalized to actin depicted in the graph (**E**). Additionally, the level of apoptosis was quantified using flow cytometry (**F**). (**G–I**) Bcl-xL has an impact on SADS-CoV replication. The ST cells, Bcl-xL-overexpressing ST cells, and ST-∆Bcl-xL cells were infected with SADS-CoV for 24 h. The SADS-CoV-N mRNA expression was detected by RT-qPCR (**G**), the viral titers were assessed using the Reed-Muench method (**H**), and the level of SADS-CoV-N expression was observed using fluorescence microscopy via anti-SADS-CoV-N mAb (**I**). Scale bars, 100 µm. The results shown are representative of three independent experiments (mean ± SD). *, *P* < 0.05; **, *P* < 0.01; ns represents no statistical difference.

To investigate whether Bcl-xL is essential for NS3-induced apoptosis, we generated the Bcl-xL-knockout ST cell line, referred to as ST-ΔBcl-xL (Fig. 5A), and assessed the impact of Bcl-xL knockout on the capability of NS3 in induction of apoptosis. First, the LDH and cellular ATPase activity were measured to rule out the possible impact of Bcl-xL depletion on the viability of ST cells. As shown in Fig. 5B and C, the knockout of Bcl-xL did not significantly affect the viability of ST cells. Immunoblotting analyses revealed that NS3 failed to induce significant cleavage of Caspase 3/9 and PARP1 in ST-ΔBcl-xL cells (Fig. 5D and E). Furthermore, flow cytometry analysis was employed to identify the apoptosis level induced by NS3 in ST-ΔBcl-xL cells. The results demonstrated that NS3 did not significantly increase apoptosis in these cells (Fig. 5F). Overall, these suggest Bcl-xL is required for NS3-mediated apoptosis.

Bcl-xL is known to inhibit apoptosis, and its overexpression has been shown to suppress the replication of viruses (16). To investigate the role of Bcl-xL in viral replication, SADS-CoV was used to infect ST cells, ST-Bcl-xL-overexpressing cells, and ST-ΔBcl-xL cells. Our findings revealed that the overexpression of Bcl-xL significantly reduced the replication efficiency of SADS-CoV, whereas the knockout of Bcl-xL enhanced the viral replication (Fig. 5G through I). These results indicate that Bcl-xL functions as an antiviral factor capable of inhibiting the replication of SADS-CoV.

### The deletion of the NS3 gene reduces the ability of SADS-CoV to induce apoptosis

**Fig 6:**
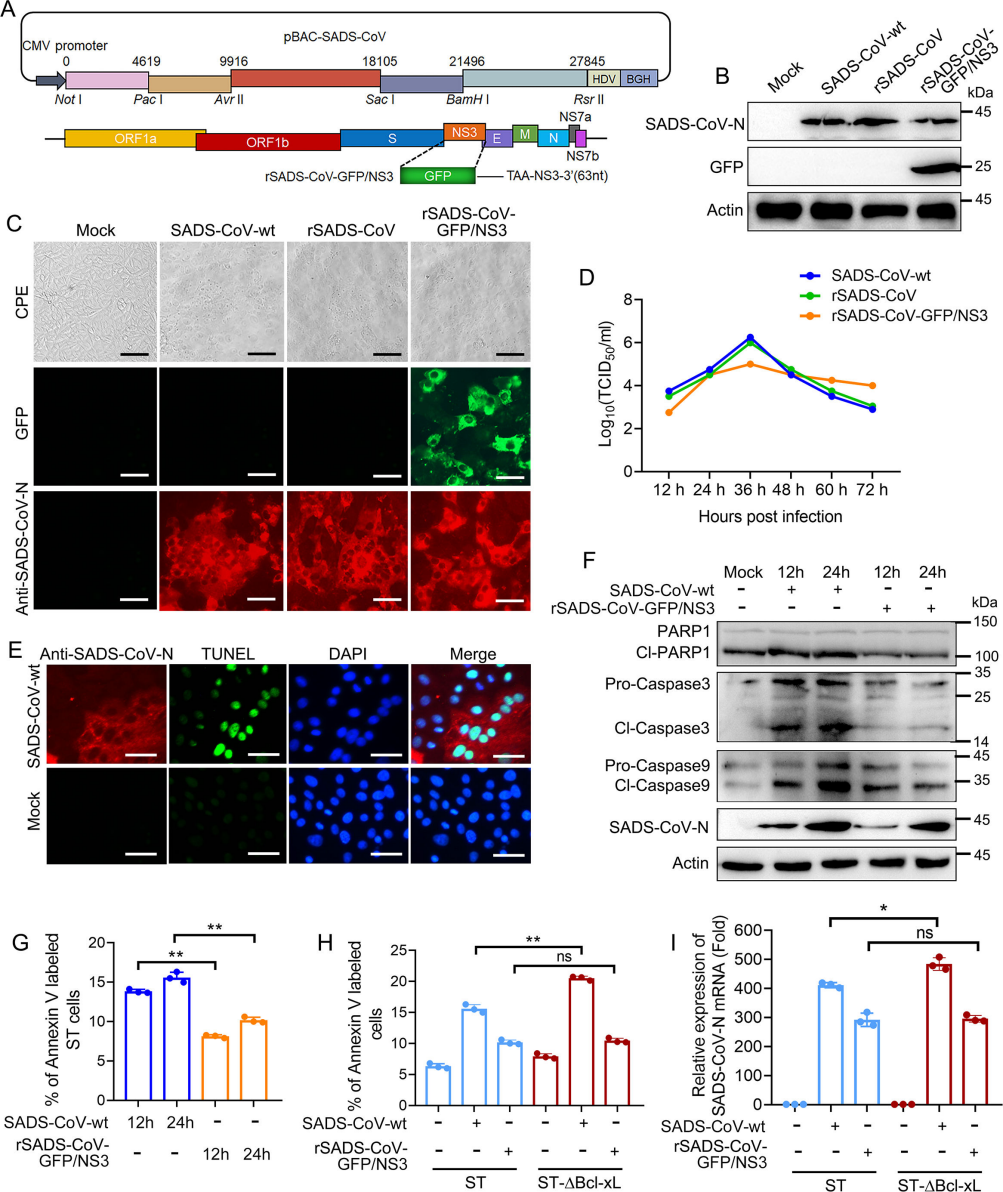
NS3 gene deletion decreased SADS-CoV-induced apoptosis. (**A**) Schematic diagram depicting the construction of the pBAC-SADS-CoV and pBAC-SADS-CoV-GFP/NS3 plasmids. (**B and C**) Recovery and identification of recombinant virus. The pBAC-SADS-CoV and pBAC-SADS-CoV-GFP/NS3 plasmids were transfected into Vero cells for 24 h, and the cytopathic effects of SADS-CoV-wt, rSADS-CoV, and rSADS-CoV-GFP/NS3 infection were observed by microscope. The expression of SADS-CoV N and GFP was detected using fluorescence microscopy and Western blotting. Scale bars, 100 µm. (**D**) Multiple-step growth curve analysis of SADS-CoV-wt, rSADS-CoV, and rSADS-CoV-GFP/NS3 in Vero cells. (**E**) ST cells were infected with SADS-CoV. The expression of SADS-CoV N was detected using IFA, and the DNA damage in the cells was detected by TUNEL staining. Scale bars, 100 µm. (**F and G**) Deletion of the NS3 gene decreased SADS-CoV-induced apoptosis. ST cells were infected with SADS-CoV or rSADS-CoV-GFP/NS3 at a MOI = 0.1 for 12 and 24 h. The cleavage of Caspase 3/9 and PARP1 in the cell lysates was determined via Western blotting (**F**). The apoptosis level was analyzed by flow cytometry (**G**). (**H and I**) ST cells and ST-∆Bcl-xL cells were respectively infected with SADS-CoV or rSADS-CoV-GFP/NS3 at a MOI = 0.1 for 24 h, and then the level of apoptosis was quantified using flow cytometry (**H**) ;the SADS-CoV-N mRNA expression was detected by RT-qPCR (**I**). The results shown are representative of three independent experiments (mean ± SD). *, *P* < 0.05; **, *P* < 0.01; ns represents no statistical difference.

To determine whether NS3 is required for SADS-CoV-induced apoptosis, we constructed an infectious clone plasmid of SADS-CoV and replaced the NS3 gene with the GFP gene through Red recombination (Fig. 6A). Subsequently, the rSADS-CoV and rSADS-CoV-GFP/NS3 viruses were rescued in Vero cells. The detection of growth characteristics showed that the rSADS-CoV was similar to that of SADS-CoV-wt, while the replication efficiency of rSADS-CoV-GFP/NS3 was significantly lower than that of its parental virus within 48 h (Fig. 6B through D). Moreover, the ST cells were infected with SADS-CoV. Subsequently, indirect immunofluorescence and TUNEL staining were employed to analyze the viral infection and apoptosis. The results demonstrated that apoptosis predominantly occurs in the virus-infected cells (Fig. 6E). Next, we compared the levels of apoptosis induced by SADS-CoV-wt and rSADS-CoV-GFP/NS3 infection. The results showed that both SADS-CoV-wt and rSADS-CoV-GFP/NS3 exhibited Caspase 3/9 activation and PARP1 cleavage at 12 and 24 hpi, but the expression level of cleaved-Caspase 3/9 and PARP1 induced by rSADS-CoV-GFP/NS3 was lower than that of SADS-CoV-wt at each time point (Fig. 6F). Concurrently, the expression levels of N proteins in rSADS-CoV-GFP/NS3-infected ST cells were also lower than those in SADS-CoV-wt-infected cells at each time point (Fig. 6F). Furthermore, the lower percentage of Annexin-V-positive cells induced by rSADS-CoV-GFP/NS3 than SADS-CoV-wt confirmed that the ability of rSADS-CoV-GFP/NS3 to induce apoptosis is weaker than SADS-CoV-wt (Fig. 6G).

Additionally, we compared the apoptosis induction of SADS-CoV-wt and rSADS-CoV-GFP/NS3 in ST and ST-ΔBcl-xL cells by flow cytometry. The results indicated that the absence of Bcl-xL significantly increased apoptosis induced by SADS-CoV-wt, whereas it did not have a substantial impact on apoptosis induced by rSADS-CoV-GFP/NS3 (Fig. 6H). Furthermore, the RT-qPCR was utilized to quantify viral replication in both cell types. Our findings reveal that the absence of Bcl-xL significantly enhanced the replication of SADS-CoV-wt, while it did not markedly influence the replication of rSADS-CoV-GFP/NS3 (Fig. 6I). Overall, these results suggest that although NS3 is not required for SADS-CoV replication, its absence adversely affects SADS-CoV-induced cell apoptosis and viral replication.

### NS3 is a virulence gene for the pathogenicity of SADS-CoV *in vivo*

**Fig 7:**
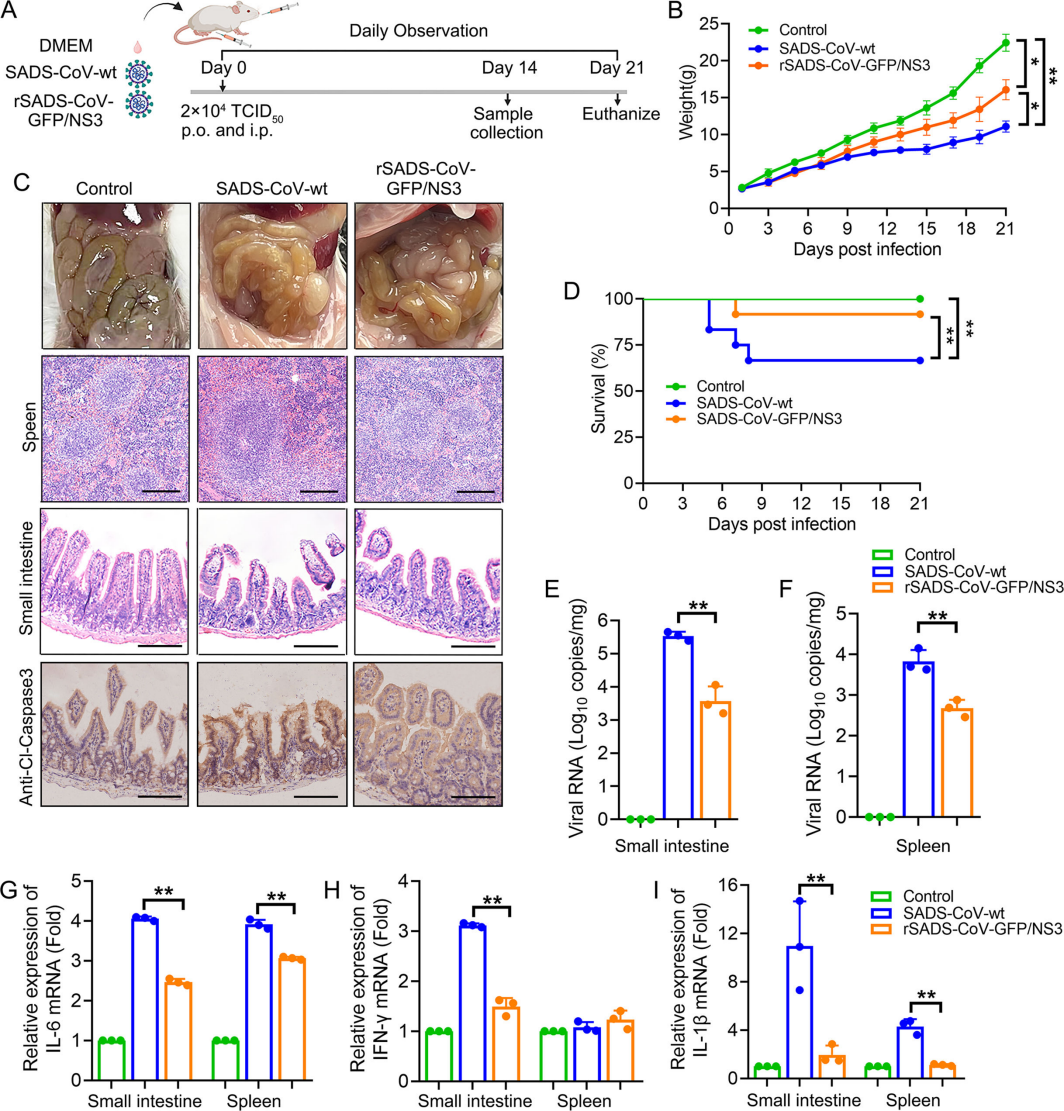
Deletion of the NS3 gene reduces pathogenicity of SADS-CoV *in vivo*. (**A**) Three- to four-day-old ICR mice were challenged orally and intraperitoneally with SADS-CoV-wt or rSADS-CoV-GFP/NS3 at a dose of 2 × 10^4^ TCID_50_ (*n* = 12). (**B**) The weight loss of mice was recorded for 21 days. (**C**) Histopathological alterations in the small intestine and spleen were identified through H&E staining. Apoptotic activity in the small intestine was assessed via immunohistochemistry employing the Cleaved-Caspase three antibody. Scale bars, 100 μm. (**D**) Survival rate. The survival of mice was recorded for 21 days. (**E and F**) Viral loads (RNA genome copies/mg) in the small intestine and spleen were evaluated by quantitative reverse transcription PCR. (**G-I**) The mRNA levels of IL-6, IFN-γ, and IL-1β in the small intestines and spleens were also evaluated by quantitative reverse transcription PCR. The results shown are representative of three independent experiments (mean ± SD). *, *P* < 0.05; **, *P* < 0.01.

The role of NS3 in the pathogenicity of SADS-CoV was further analyzed *in vivo* by challenging 3- to 4-day-old SPF ICR mice with SADS-CoV-wt or rSADS-CoV-GFP/NS3 (Fig. 7A). Daily monitoring revealed that mice infected with SADS-CoV-wt experienced diarrhea and weight loss, whereas those infected with rSADS-CoV-GFP/NS3 exhibited only slight weight loss and diarrhea (Fig. 7B). In comparison to the control group, the mice in the SADS-CoV-wt group displayed significant tissue damage; the intestines were gas-filled and transparent; the intestinal villi were atrophied, broken, and detached; the number of lymphocytes in the spleen increased; and the number of germinal centers in the spleen expanded (Fig. 7C). Conversely, these pathological changes were minimal in the rSADS-CoV-GFP/NS3 group (Fig. 7C). Notably, the Cleaved-Caspase 3-positive cells in the small intestines were significantly reduced in rSADS-CoV-GFP/NS3-infected mice, compared with those infected with SADS-CoV-wt (Fig. 7C). As shown in Fig. 7D, four mice (*n* = 12) challenged with SADS-CoV-wt died within 7 dpi, while only one mouse died (*n* = 12) in the group challenged with rSADS-CoV-GFP/NS3. The viral loads in the small intestine and spleen tissues of mice challenged with SADS-CoV-wt were significantly higher than those in the group challenged with rSADS-CoV-GFP/NS3 (Fig. 7E and F), which indicated that NS3 deletion caused a decrease in the replication efficiency of SADS-CoV in mice. Additionally, the mRNA levels of inflammatory cytokines (IFN-γ, IL-1β, and IL-6) in the small intestine and spleen of mice indicated a reduced inflammatory response in the rSADS-CoV-GFP/NS3 group compared with the SADS-CoV-wt group (Fig. 7G through I). Together, these findings suggest that NS3 gene deletion significantly reduces SADS-CoV-induced intestinal apoptosis and attenuates the pathogenicity of SADS-CoV in mice.

## DISCUSSION

**Fig 8:**
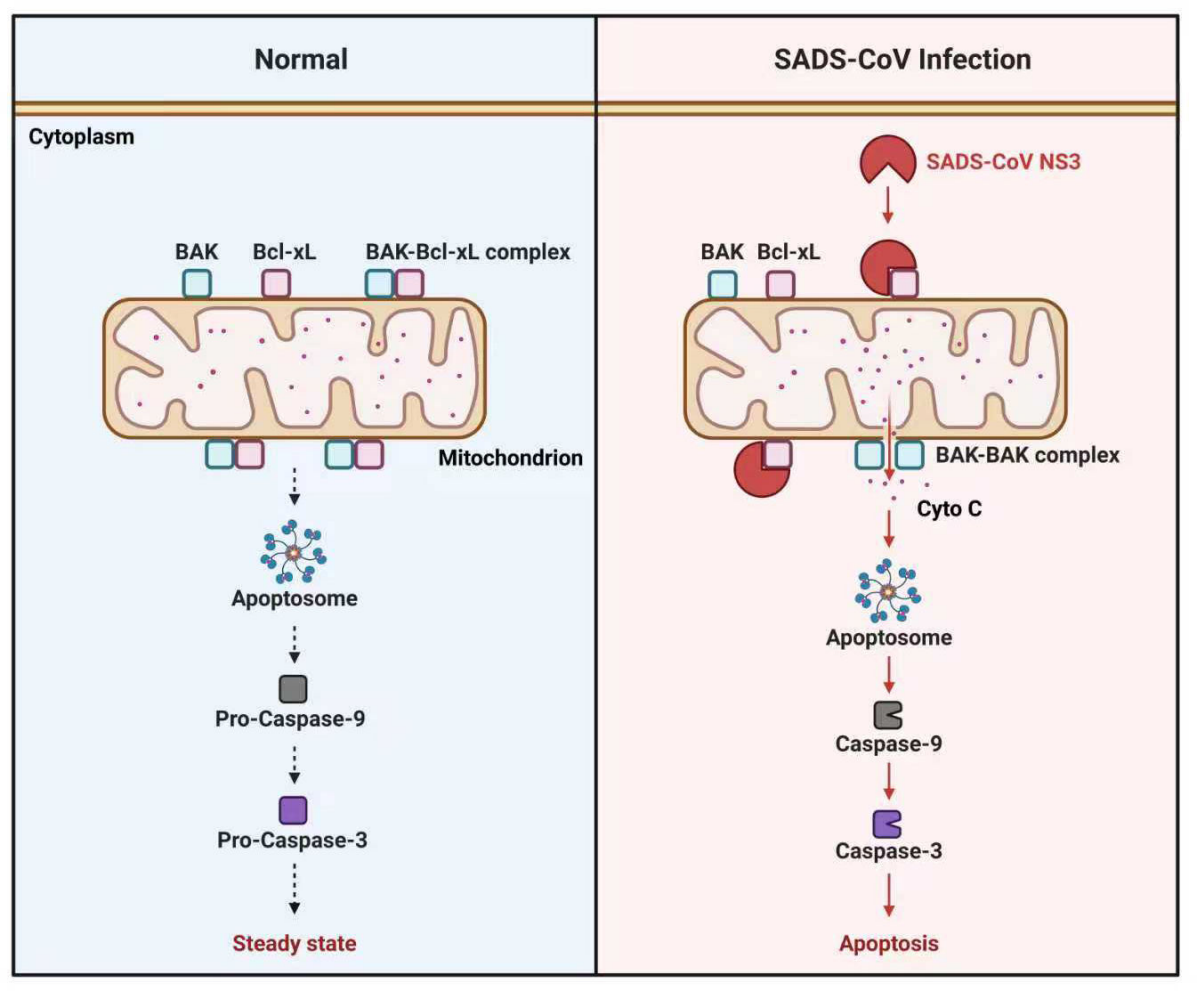
Schematic representation of SADS-CoV NS3-induced apoptosis. In the steady state, the pro-apoptotic protein BAK and the anti-apoptotic protein Bcl-xL form dimers within the mitochondria to sustain cellular homeostasis. During SADS-CoV infection, virus-encoded NS3 competitively binds to Bcl-xL within the mitochondria, disrupting the Bcl-xL-BAK complex and facilitating BAK oligomerization. Subsequently, the integrity of the mitochondrial membrane is damaged, leading to Cyto C release, which subsequently promotes the formation of apoptotic bodies and DNA fragmentation. Ultimately, this cascade leads to the activation of apoptotic effectors Caspase 3/9, culminating in apoptosis of the infected cells. This apoptotic pathway enhances both the replication and pathogenicity of SADS-CoV.

Apoptosis can disrupt the structure and integrity of the intestinal mucosa and lead to associated acute diarrhea in intestines infected with porcine coronavirus (17). Coronaviruses have evolved mechanisms to induce apoptosis, facilitating the production of progeny viruses or the establishment of persistent infections (18). As reported, SADS-CoV can induce caspase-dependent extrinsic and intrinsic apoptosis (12). However, the specific viral proteins responsible for SADS-CoV-induced apoptosis and the associated molecular mechanisms have yet to be fully elucidated. Coronavirus accessory proteins play a crucial role in interactions between the virus and host, affecting the host’s apoptosis and antiviral response, and ultimately impacting viral pathogenesis. These proteins, such as SARS-CoV ORF3a, ORF7a, and ORF8a, primarily activate the intrinsic pathway of apoptosis (19). A recent study revealed that SADS-CoV NS7a interacted with apoptosis-inducing factor mitochondria associated 1 (AIFM1) to activate Caspase3, ultimately leading to apoptosis (13). However, in this study, it was found that the apoptosis induced by NS3 was also pronounced. Furthermore, our findings demonstrated that SADS-CoV NS3 could localize to the mitochondria, where it combined with Bcl-xL and disrupted the Bcl-xL-BAK complex, leading to increased BAK homo-oligomerization. Subsequently, the mitochondrial membrane potential was destroyed to promote the release of Cyto C, resulting in the activation of the intrinsic pathway of apoptosis (Fig. 8).

The regulation of the intrinsic apoptosis pathway is mainly dependent on the Bcl-2 protein family. As a member of this family, Bcl-xL plays an important role in inhibiting apoptosis and maintaining homeostasis during viral infection (20, 21). For example, Bcl-xL inhibited the apoptosis induced by SARS-CoV ORF7a protein (22). The apoptosis induced by the SARS-CoV E protein in Jurkat T cells could be suppressed through the overexpression of Bcl-xL (23). Bcl-xL contains four functionally homologous hydrological regions (BH1, BH2, BH3, and BH4), which form an elongated hydrophobic cleft to combine with other apoptotic proteins (24). Among the four regions, the BH3 region of Bcl-xL is crucial for its interaction with the proapoptotic protein BAK (15). Bcl-xL binds to BAK to form the Bcl-xL-BAK complex, which reduces the BAK homo-oligomerization and maintains mitochondrial apoptosis at normal levels. Notably, some viral proteins could interact with the BH3 region of Bcl-xL, thereby impeding the binding site on Bcl-xL and interfering with its antiapoptotic activity. For example, the SARS-CoV E protein could interact with the BH3 domain of Bcl-xL, which served as the underlying mechanism for E protein-induced apoptosis (23). The hepatitis B virus X protein (HBX) induces apoptosis through its interaction with the BH3-like motif of Bcl-xL (25). In our study, apoptosis induced by NS3 was also markedly diminished in cells overexpressing Bcl-xL, whereas there was no notable increase in NS3-induced apoptosis in ST-∆Bcl-xL cells. Notably, we demonstrated that NS3 can bind to the BH3 region of Bcl-xL, thereby effectively disrupting the formation of Bcl-xL-BAK complexes. These findings demonstrate that Bcl-xL is required for NS3-induced apoptosis, and the apoptosis occurred dependently on the interaction between Bcl-xL and NS3.

Research has shown that coronavirus accessory protein-induced apoptosis plays a pivotal role in viral replication. The SARS-CoV-2 ORF8 can induce apoptosis, and its absence suppresses viral replication and reduces inflammation in SARS-CoV-2-infected mice (26). The ORF3a of SARS-CoV and SARS-CoV-2 also induces apoptosis and plays crucial roles in viral virulence in mouse models (27). To further elucidate the roles of NS3 in SADS-CoV replication and pathogenicity, we generated recombinant SADS-CoV with the NS3 gene substituted by GFP. Unfortunately, we could not obtain recombinant SADS-CoV with NS3 deletion alone, which suggests that the complexities involved in the transcriptional regulation of the SADS-CoV NS3 gene remain to be resolved. Upon deletion of the NS3 gene, virus-induced apoptosis was significantly reduced. Meanwhile, the expression levels of N proteins in rSADS-CoV-GFP/NS3-infected ST cells were lower than those in SADS-CoV-wt-infected cells. Furthermore, the virus growth curve assay also showed that SADS-CoV-wt grew better than rSADS-CoV-GFP/NS3 within 48 hpi, where the titer of SADS-CoV-wt peaked at 36 hpi, with more than 1 log higher than rSADS-CoV-GFP/NS3. This suggests that NS3-induced apoptosis contributed to the replication of SADS-CoV. At the late stage (after 48 hpi) in the virus growth curve, the titers of rSADS-CoV-GFP/NS3 became higher than those of SADS-CoV-wt, potentially due to most of SADS-CoV-wt-infected cells dying, while a considerable number of rSADS-CoV-GFP/NS3-infected cells remained to support the virus growth.

Additionally, the mouse model was reported to be an economical choice for investigation of SADS-CoV pathogenicity. The suckling mice were susceptible to SADS-CoV after challenge via various routes, including oral (o.p.), intragastric (i.g.), intraperitoneal (i.p.), and intracranial (i.c.) routes (2, 5, 28). Although the intracranial (i.c.) inoculation with SADS-CoV at a dose of 8 × 10^5^ TCID_50_ could lead to 100% mortality (28), it is not a natural infection way. In the other challenge routes, the mortality rates of mice were under 70% with the same dose (2, 28). In our study, the suckling mice were challenged orally and intraperitoneally with SADS-CoV at a dose of 2 × 10^4^ TCID_50_ lower than previously reported. The SADS-CoV-wt-infected mice exhibited significant watery diarrhea and weight loss, with a morbidity rate of 100% and a mortality rate of 30%, thereby indicating that the SADS-CoV-mouse model was established successfully. In comparison, the mice infected with rSADS-CoV-GFP/NS3 exhibited mild weight loss, low mortality, and reduced levels of intestinal cell apoptosis. The results suggest that the NS3 plays a critical role in the pathogenicity of SADS-CoV.

In summary, this study is the first to elucidate the role of the NS3 protein in SADS-CoV. The NS3 protein interacts with the antiapoptotic protein Bcl-xL and disrupts the Bcl-xL-BAK complex, thereby inducing mitochondrion-mediated apoptosis. Bcl-xL serves as an antiviral factor in inhibiting SADS-CoV replication. Furthermore, NS3 has been identified as a significant contributor to the virulence of SADS-CoV, with its apoptotic effects being closely associated with viral propagation and disease severity. The results of this study will greatly contribute to understanding the diverse roles of accessory proteins of bat-origin coronaviruses and provide insights into how to develop effective control strategies against virus infection across species boundaries.

## Data Availability

All data sets generated and/or analyzed during the current study are presented in the article and the accompanying supplemental material or are available from the corresponding author upon reasonable request.
